# Inhibition of Mitochondria- and Endoplasmic Reticulum Stress-Mediated Autophagy Augments Temozolomide-Induced Apoptosis in Glioma Cells

**DOI:** 10.1371/journal.pone.0038706

**Published:** 2012-06-22

**Authors:** Chien-Ju Lin, Chin-Cheng Lee, Yung-Luen Shih, Chien-Huang Lin, Sheng-Hao Wang, Thay-Hsiung Chen, Chwen-Ming Shih

**Affiliations:** 1 Graduate Institute of Medical Sciences, College of Medicine, Taipei Medical University, Taipei, Taiwan; 2 Department of Biochemistry, College of Medicine, Taipei Medical University, Taipei, Taiwan; 3 Department of Pathology and Laboratory Medicine, Shin Kong Wu Ho-Su Memorial Hospital, Taipei, Taiwan; 4 School of Medical Laboratory Science and Biotechnology, College of Medicine, Taipei Medical University, Taipei, Taiwan; 5 Division of Cardiovascular Surgery, Department of Surgery, Cathay General Hospital, Taipei, Taiwan; 6 Traditional Herbal Medicine Research Center, Taipei Medical University Hospital, Taipei, Taiwan; 7 Center for Reproductive Medicine and Sciences, Taipei Medical University Hospital, Taipei, Taiwan; Rush University Medical Center, United States of America

## Abstract

Autophagy is a crucial process for cells to maintain homeostasis and survival through degradation of cellular proteins and organelles, including mitochondria and endoplasmic reticula (ER). We previously demonstrated that temozolomide (TMZ), an alkylating agent for brain tumor chemotherapy, induced reactive oxygen species (ROS)/extracellular signal-regulated kinase (ERK)-mediated autophagy to protect glioma cells from apoptosis. In this study, we investigated the role of mitochondrial damage and ER stress in TMZ-induced cytotoxicity. Mitochondrial depolarization and mitochondrial permeability transition pore (MPTP) opening were observed as a prelude to TMZ-induced autophagy, and these were followed by the loss of mitochondrial mass. Electron transport chain (ETC) inhibitors, such as rotenone (a complex I inhibitor), sodium azide (a complex IV inhibitor), and oligomycin (a complex V inhibitor), or the MPTP inhibitor, cyclosporine A, decreased mitochondrial damage-mediated autophagy, and therefore increased TMZ-induced apoptosis. TMZ treatment triggered ER stress with increased expression of GADD153 and GRP78 proteins, and deceased pro-caspase 12 protein. ER stress consequently induced autophagy through c-Jun N-terminal kinases (JNK) and Ca^2+^ signaling pathways. Combination of TMZ with 4-phenylbutyrate (4-PBA), an ER stress inhibitor, augmented TMZ-induced cytotoxicity by inhibiting autophagy. Taken together, our data indicate that TMZ induced autophagy through mitochondrial damage- and ER stress-dependent mechanisms to protect glioma cells. This study provides evidence that agents targeting mitochondria or ER may be potential anticancer strategies.

## Introduction

Autophagy is a process by which long-lived proteins and organelles in the cytoplasm are degraded [1]. It is characterized by the formation of autophagic vacuoles in the cytoplasm, called autophagosomes. The fusion of autophagosome to lysosome generates an autolysosome structure. Cellular components embedded in autophagosomes are degraded by lysosomal enzymes to provide materials which are used in bio-synthetic reactions and ATP production [2]. Thus, it is essential that cells undergo autophagy to maintain their vitality in difficult situations, including starvation, viral infection, and some diseases such as neurodegenerative diseases, cancers, and aging [3].

Damaged mitochondria are also removed through autophagy in cells [4]. If autophagy is eliminated by Atg 7 deletion, mitochondrial function is reduced and the reactive oxygen species (ROS) level increases, resulting in physiological impairment [5]. In Parkinson’s disease, accumulation of defective mitochondria causes neuronal cell death [6]. In addition, accumulation of damaged mitochondria may lead to tumorigenesis; therefore, dysfunctional mitochondria should be eliminated in physiologic conditions [7]. An oral alkylating agent, temozolomide (TMZ), is used in clinical chemotherapy for patients with glioblastoma for its good absorption and penetration through the blood-brain barrier [8]. In our previous study, we revealed that TMZ induces the generation of ROS and extracellular signal-regulated kinase (ERK) activation, which consequently leads to protective autophagy in glioma cells [9]. The source of ROS is mainly from mitochondria due to operation of the respiratory chain [10], and excessive ROS can damage mitochondria and result in autophagy or apoptosis [10]–[12]. However, whether TMZ treatment can cause mitochondrial damage and the relationship between mitochondria and TMZ-induced autophagy and apoptosis are still unclear.

Endoplasmic reticula (ER) are organelles in which secreted and membrane proteins are modified, folded, and assembled [13]. When ER experience adverse situations, such as nutrient deprivation, hypoxia, unbalance of calcium homeostasis, failure of posttranslational modifications, and increased protein synthesis, the accumulation of unfolded proteins are increased, that called ER stress [14]. ER stress triggers the unfolded protein response (UPR) to reduce protein synthesis and increase the capacity of protein folding. During the UPR, the ER chaperone, glucose-regulated protein 78 (GRP78), disassociates from three signaling receptors, pancreatic ER kinase (PKR)-like ER kinase (PERK), activating transcription factor 6 (ATF6) and inositol-requiring enzyme 1 (IRE1), thus transducing death or survival signals [14]–[16]. Therefore, ER stress may play a prosurvival or proapoptotic role. Others’ and our previous studies revealed that severe ER stress triggers apoptotic cell death [17], [18]. Studies also indicated that ER stress can induce autophagy [19], [20]. Therefore, the role of ER stress in determining the fate of cells treated with TMZ is worth investigating.

In this report, we investigated the role of mitochondria and ER in TMZ-treated glioma cells. Our results showed that TMZ induced mitochondrial depolarization and the opening of mitochondrial permeability transition pores (MPTP), and subsequently triggered autophagy to diminish mitochondrial mass in U87 MG malignant glioma cells. Inhibition of the electron transport chain (ETC) by rotenone, sodium azide, or oligomycin suppressed the percentage of cells undergoing autophagy through reducing mitochondrial damage, while TMZ-induced apoptosis was augmented. TMZ also induced ER stress-mediated autophagy and apoptosis through c-Jun N-terminal kinases (JNK) and Ca^2+^ signaling pathways. However, the ER stress inhibitor, 4-phenylbutyrate (4-PBA), further increased the cytotoxicity of TMZ. Our results offer an inference that drugs targeting mitochondria or ER may improve chemotherapy for brain tumors.

## Results

### TMZ Induces Mitochondrial Depolarization, the Opening of MPTP, and the Loss of Mitochondrial Mass

**Figure 1:**
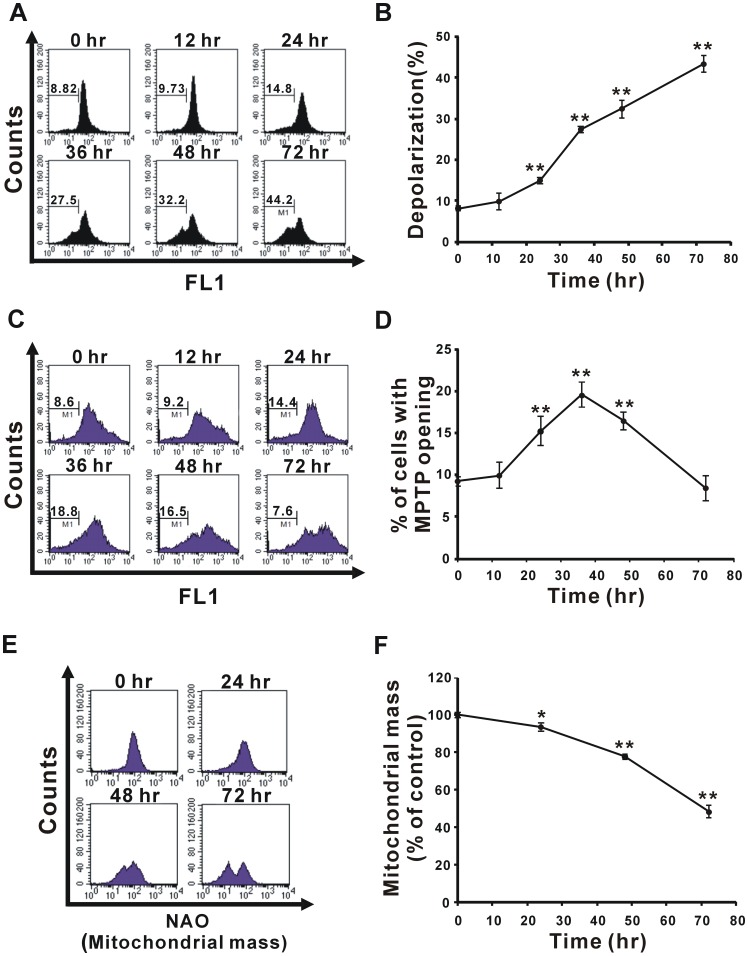
TMZ induces mitochondrial depolarization, MPTP opening, and loss of mitochondrial mass. U87 MG cells were treated with 400 µM TMZ for the indicated time course. The mitochondrial membrane potential (A and B), MPTP (C and D), and mitochondrial mass (E and F) were measured using flow cytometry with rhodamine 123, calcein AM/CoCl_2_, and NAO staining, respectively. Data presented in panels (A), (C), and (E) are representative of three independent experiments, and their statistical results are presented as the mean ± SD in panels (B), (D), and (F), respectively. *p<0.05, **p<0.01 vs. each respective control.

In our previous study, we demonstrated that 100–500 µM TMZ suppressed glioma cell proliferation in a dose-dependent manner, and the IC_50_ of TMZ was calculated to be 397.2 µM in U87 MG cells [9]. Therefore, a dose of 400 µM TMZ was selected for use in the following experiments to study the role of mitochondria in TMZ-induced cytotoxicity. To investigate whether mitochondrial damage was induced by TMZ, we analyzed the characteristics of mitochondrial damage, such as mitochondrial depolarization, MPTP opening, and the change of mitochondrial mass. First, the mitochondrial membrane potential (ΔΨm) was measured by rhodamine123 staining after treatment with 400 µM TMZ for 0–72 h. A significant depolarization of mitochondria was observed at 24 h and sustained to 72 h (Figure 1A and B). Following this line, we further analyzed the degree of MPTP opening on a flow cytometer using calcein AM and CoCl_2_ co-labeling (see “Materials and Methods” for details) after treating glioma cells with 400 µM TMZ for various time courses. The results demonstrated that the proportion of TMZ-treated glioma cells with loss of calcein fluorescence increased from 24 h and had reached a maximum at 36 h (Figure 1C and D), suggesting that MPTP opening was induced by TMZ treatment. The 10-N-nonyl-acridine orange (NAO) staining indicated that mitochondrial mass declined after treatment with TMZ for 24 h and had almost decreased to 50% at 72 h (Figure 1E and F). These results demonstrate that TMZ induced mitochondrial damage, including the loss of ΔΨm and the opening of MPTP, and resulted in a decrease of mitochondrial mass, suggesting that mitochondrial damage might participate in TMZ-induced cell death.

### TMZ-induced Mitochondrial Depolarization and Loss of Mitochondrial Mass are Reversed by ETC Inhibitors

**Figure 2:**
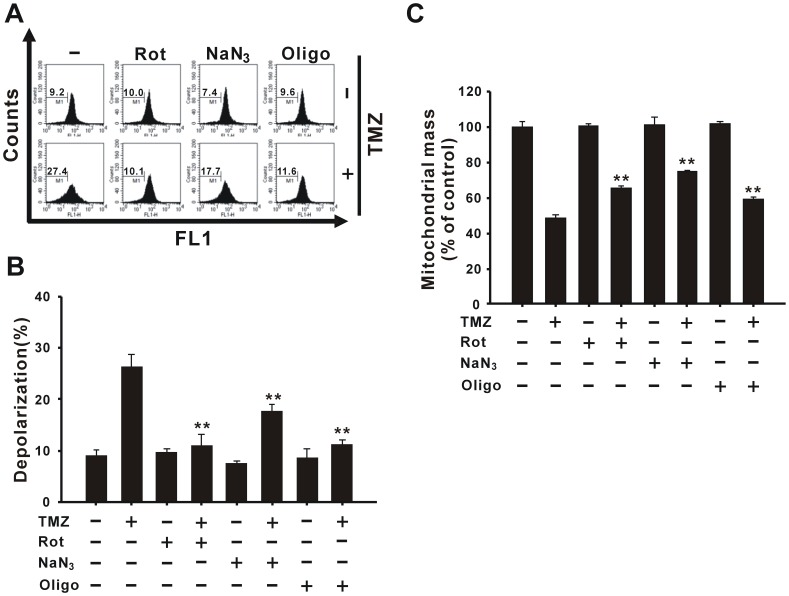
The ETC inhibitors reverse TMZ-induced mitochondrial depolarization and the loss of mitochondrial mass. (A and B) U87 MG cells were pre-treated with or without 20 nM rotenone, 150 µM sodium azide, or 1 nM oligomycin for 1 h followed by incubation with 400 µM TMZ for 36 h, and then the mitochondrial membrane potential was analyzed using flow cytometry with rhodamine 123. (C) Mitochondrial mass was detected with NAO staining after the same treatment for 72 h. Data presented in panel (A) are representative of three independent experiments, and their statistical results are presented as the mean ± SD in panel (B). Rot, rotenone; NaN_3_, sodium azide; Oligo, oligomycin. **p<0.01 vs. each respective TMZ group.

We next analyzed the influence of mitochondrial damage on TMZ-induced autophagy and/or apoptosis. ETC inhibitors, such as rotenone (a complex I inhibitor), sodium azide (a complex IV inhibitor), and oligomycin (a complex V inhibitor), were employed to investigate their effects on mitochondria. These ETC inhibitors were administered with or without 400 µM TMZ in glioma cells for 36 h, and the status of mitochondria was monitored. As shown in Figure 2A and B, TMZ-induced collapse of ΔΨm was abolished by all of these ETC inhibitors. Remarkably, rotenone and oligomycin totally reversed TMZ-induced mitochondrial depolarization. In addition, the combination of ETC inhibitors and TMZ increased mitochondrial mass compared to the TMZ group after 72 h of treatment (Figure 2C). These data reveal that the ETC plays crucial roles in the cytotoxicity of TMZ toward glioma cells by inducing mitochondrial damage.

### ETC Inhibitors Decrease the ROS Generation

**Figure 3:**
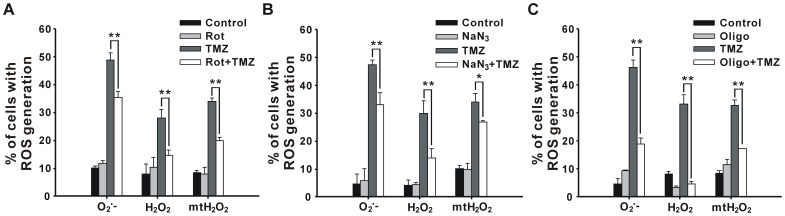
ETC inhibitors suppress TMZ-induced ROS generation. U87 MG cells were pretreated with or without 20 nM rotenone, 150 µM sodium azide, or 1 nM oligomycin for 1 h followed by incubation with 400 µM TMZ for 36 h, and ROS levels were analyzed by HEt, DCFH-DA, and DHR123 staining using flow cytometry. Rot, rotenone; NaN_3_, sodium azide; Oligo, oligomycin. Results are presented as the mean ± SD. *p<0.05, **p<0.01 vs. each respective TMZ group.

Our previous study demonstrated that TMZ induces ROS generation in U87 MG cells [9]. Since ROS is demonstrated to mainly be generated from the respiratory chain [10], we next used ETC inhibitors to analyze whether ROS levels were influenced. After U87 MG cells were treated with individual ETC inhibitors and 400 µM TMZ for 36 h, intracellular O_2_^−^, H_2_O_2_, and mitochondrial H_2_O_2_ (mtH_2_O_2_) levels were measured using the specific dyes dihydroethidium (HEt), 2,7-dihydrodichlorofluorescein diacetate (DCFH-DA), and dihydrorhodamine123 (DHR123), respectively. As shown in Figure 3, TMZ-induced ROS accumulation, including intracellular O_2_^−^, H_2_O_2_, and mtH_2_O_2_, were all suppressed by the addition of rotenone (Figure 3A), sodium azide (Figure 3B), and oligomycin (Figure 3C). These results suggest that mitochondrial damage might be one of the major causes of TMZ-induced ROS generation.

### Decreasing TMZ-induced Autophagy by ETC Inhibitors Augments Apoptosis and Cell Death

**Figure 4:**
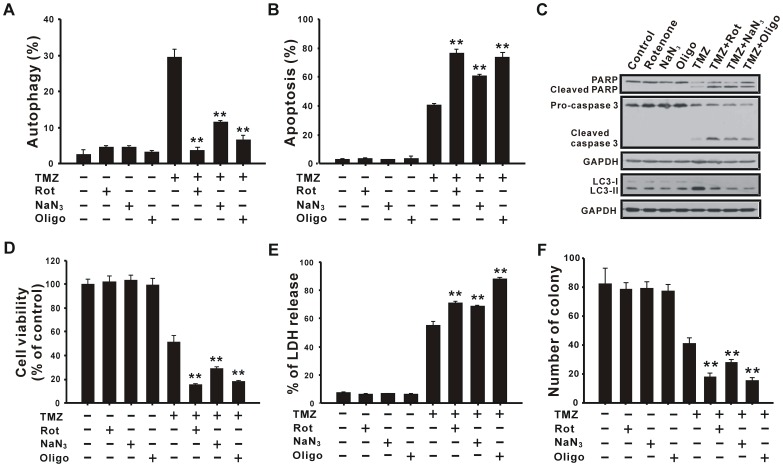
ETC inhibitors reduce TMZ-induced autophagy and increase apoptosis and cell death. U87 MG cells were treated with or without 20 nM rotenone, 150 µM sodium azide, or 1 nM oligomycin for 1 h followed by incubation with 400 µM TMZ for another 72 h. Autophagy (A) and apoptosis (B) were assessed as described in “Materials and Methods”. (C) U87 MG cells were pretreated with ETC inhibitors such as rotenone, sodium azide, and oligomycin for 1 h, followed by treatment with TMZ for 36 h. Cell lysates were analyzed by immunoblotting using antibodies against PARP, caspase 3, LC3, and GAPDH. GAPDH was used as an internal control to normalize the amount of proteins applied in each lane. Cells were treated as in (A) and were analyzed by MTT assay (D), LDH release assay (E), and colony formation assay (F). Results are presented as the mean±SD. Rot, rotenone; NaN_3_, sodium azide; Oligo, oligomycin. **p<0.01 vs. each respective TMZ group.

The above ETC inhibitors were used to investigate the participation of mitochondrial damage in TMZ-induced autophagy. As shown in Figure 4A and C, ETC inhibitors, including rotenone, sodium azide, and oligomycin, reduced TMZ-induced autophagy and microtubule-associated protein light chain 3 (LC3) processing. On the contrary, the percentage of cells undergoing TMZ-induced apoptosis and cleavage of caspase 3 and poly (ADP-ribose) polymerase (PARP) increased with the addition of ETC inhibitors (Figure 4B and C), which resulted in reduced cell proliferation (Figure 4D) and increased lactate dehydrogenase **(**LDH) release (Figure 4E) compared to the TMZ-alone group. Furthermore, colony formation assay was performed to investigate the long-term cytotoxic effect. As shown in Figure 4F, the number of colonies further decreased after cells were treated with TMZ and ETC inhibitors. These data suggest that TMZ induces mitochondrial damage in U87 MG cells, which was followed by protective autophagy to rescue cells from apoptosis. This finding is correlated with our previous report that scavenging of TMZ-triggered ROS bursts resulted in a decrease of protective autophagy and an increase of apoptotic cell death [9]. Therefore, it seems that mitochondrial damage-mediated autophagy is crucial for cell survival. To confirm the concept, the autophagy inducer, rapamycin, was combined with ETC inhibitors and TMZ to treat U87 MG cells for 72 h, and then the cell viability was observed to have increased (Figure S1), suggesting that mitochondrial damage-mediated autophagy plays a crucial role in protecting cells from death.

### TMZ Induces Mitochondria-mediated Autophagy, which is Essential to Decrease Mitochondrial Mass

**Figure 5:**
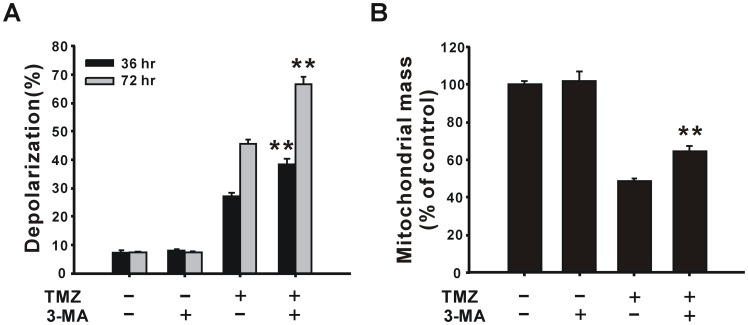
Effects of the autophagy inhibitor, 3-MA on the mitochondrial membrane potential and mitochondrial mass. U87 MG cells were pre-treated with or without 2 mM 3-MA for 1 h followed by incubation with 400 µM TMZ for 36 h or 72 h. The mitochondrial membrane potential (A) and mitochondrial mass (B) were analyzed using flow cytometry with rhodamine 123 and NAO staining, respectively. Results are presented as the mean ± SD. **p<0.01 vs. each respective TMZ group.

To reveal if mitochondrial damage is a cause or consequence of autophagy, the autophagy inhibitor, 3-methyladenine (3-MA), was administrated, which resulted in augmentation of TMZ-induced mitochondrial depolarization (Figure 5A). On the contrary, the addition of 3-MA increased mitochondrial mass compared to TMZ alone (Figure 5B). These results suggest that mitochondrial depolarization is a prelude to autophagy, and autophagy is possibly a pivotal pathway for degrading damaged mitochondria and eventually to losing mitochondrial mass after TMZ treatment. Moreover, if cells cannot undergo autophagy, mitochondrial damage will become even worse. In fact, in our previous report [9], addition of 3-MA suppresses TMZ-induced autophagy and increases TMZ-induced apoptosis in glioma cells, suggesting TMZ induced a protective autophagy.

### The Opening of MPTP Participates in Autophagy

**Figure 6:**
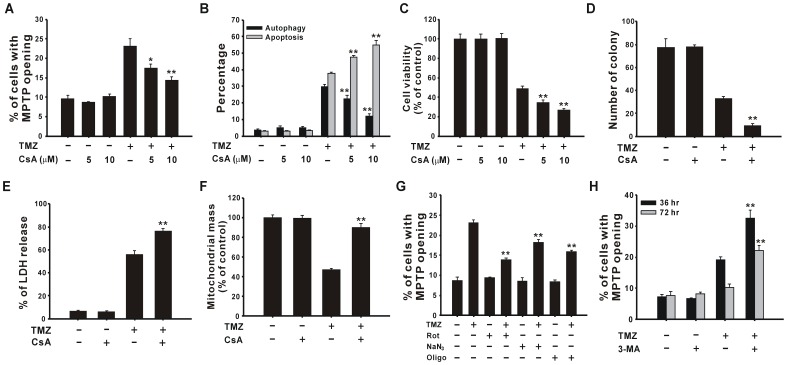
MPTP is involved in TMZ-induced autophagy. U87 MG cells were pretreated with 5 or 10 µM CsA for 1 h, followed by treatment with 400 µM TMZ for another 36 h to analyze MPTP opening, or for another 72 h to determine the percentages of cells undergoing autophagy and apoptosis (B). Cells treated with 10 µM CsA and 400 µM TMZ were analyzed by MTT assay (C) or colony formation assay (D). LDH release (E) and mitochondrial mass (F) were detected after TMZ treatment with 10 µM CsA for 72 h. The ETC inhibitors rotenone, sodium azide, and oligomycin (G) or 3-MA (H) were combined with 400 µM TMZ for 36 h, and MPTP was detected. Results are presented as the mean ± SD. Rot, rotenone; NaN_3_, sodium azide; Oligo, oligomycin. **p<0.01 vs. each respective TMZ group.

The opening of MPTP was diminished by the MPTP inhibitor, cyclosporin A (CsA) (Figure 6A). To investigate the role of MPTP in TMZ-induced cytotoxicity, CsA was administrated to U87 MG cells with 400 µM TMZ for 72 h, and ratios of cells undergoing autophagy and apoptosis were detected. As revealed in Figure 6B, CsA reduced TMZ-induced autophagy and increased TMZ-induced apoptosis. Furthermore, short-term cell viability (Figure 6C) and long-term colony formation (Figure 6D) were lower in CsA and TMZ-treated cells than in those treated with TMZ only. The LDH release assay also indicated increased cytotoxicity after combined treatment with TMZ and CsA (Figure 6E). The results suggest that MPTP opening can induce protective autophagy after TMZ treatment. However, the addition of CsA reversed the TMZ-induced loss of mitochondrial mass (Figure 6F). Moreover, MPTP opening was suppressed by adding ETC inhibitors, including rotenone, sodium azide, and oligomycin (Figure 6G), which implies that mitochondrial damage may occur prior to TMZ-induced MPTP opening which resulted in autophagy. However, inhibition of autophagy by 3-MA aggravated the opening of MPTP (Figure 6H). Taken together, these results demonstrate that TMZ-induced mitochondrial damage may cause MPTP opening, which results in the process of autophagy in U87 MG glioma cells.

### ER Stress Participates in TMZ-induced Autophagy

**Figure 7:**
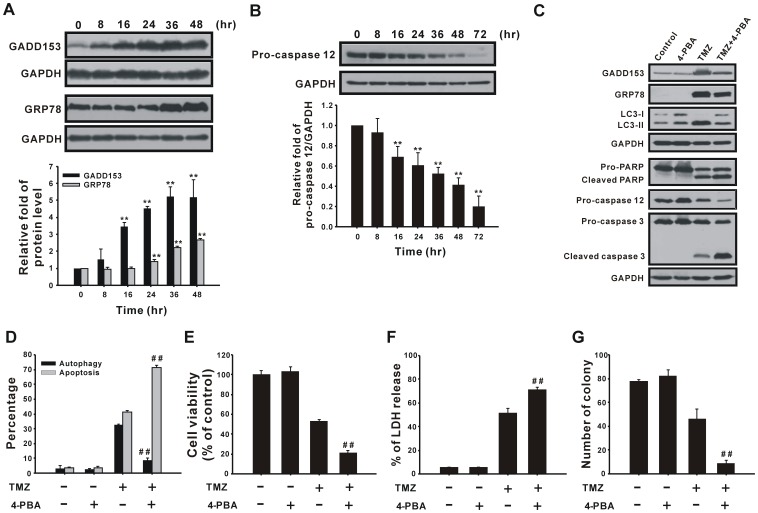
TMZ triggers ER stress to induce autophagy. (A and B) U87 MG cells were treated with 400 µM TMZ for the indicated time periods and then analyzed by immunoblotting with anti-GADD153, anti-GRP78, anti-GAPDH (A), and anti-caspase 12 (B) antibodies. (C) U87 MG cells were pretreated with 10 mM 4-PBA for 1 h, followed by treatment with TMZ for 36 h (for analysis of GRP78, GADD153, and LC3) or 72 h (for analysis of PARP, caspase 12, and caspase 3). Cell lysates were analyzed by immunoblotting. GAPDH was used as an internal control to normalize the amount of proteins applied in each lane. (D) U87 MG cells were treated as in (C) for 72 h to determine percentages of cells undergoing autophagy and apoptosis, as well as the cell viability (E) and percent of LDH release (F). (G) Long-term viability of cells after TMZ and 4-PBA co-treatment was measured by colony formation assay. Results are presented as the mean ± SD. **p<0.01 vs. each respective control. ^##^ *p*<0.01 vs. each respective TMZ group.

To investigate whether ER stress is involved in TMZ-induced autophagy and/or apoptosis, cells were treated with 400 µM TMZ for various time courses, and protein levels of GADD153, GRP78, and caspase 12, hallmarks of ER stress, were detected using immunoblotting. As shown in Figure 7A, GADD153 and GRP78 protein levels increased after treatment with TMZ and were suppressed by the ER stress modulator, 4-PBA (Figure 7C). In addition, TMZ treatment also decreased the protein level of pro-caspase 12 (Figure 7B), suggesting that ER stress may trigger apoptosis through caspase 12 activation. To further study the role of TMZ-induced ER stress, we used 4-PBA and TMZ to co-treat cells and then detected the percentages of cells undergoing autophagy and apoptosis. TMZ-induced autophagy and LC3 protein processing were reduced by 4-PBA (Figure 7C and D, black column). Parallel experiments indicated that cleavages of caspase 3, caspase 12, and PARP and the percentage of apoptosis had increased (Figure 7C and D, gray column), resulting in a decrease of cell viability (Figure 7E) and an increase of LDH release ratio (Figure 7F). Moreover, long-term colony formation was also suppressed by the addition of 4-PBA. According to these results, we suggest that TMZ-induced ER stress participates in the process of protective autophagy and apoptosis. However, our results indicate that protective autophagy plays a more important role in determining a cell’s fate.

### TMZ Treatment Increases the Calcium Concentration and Activates JNK to Induce Autophagy

**Figure 8:**
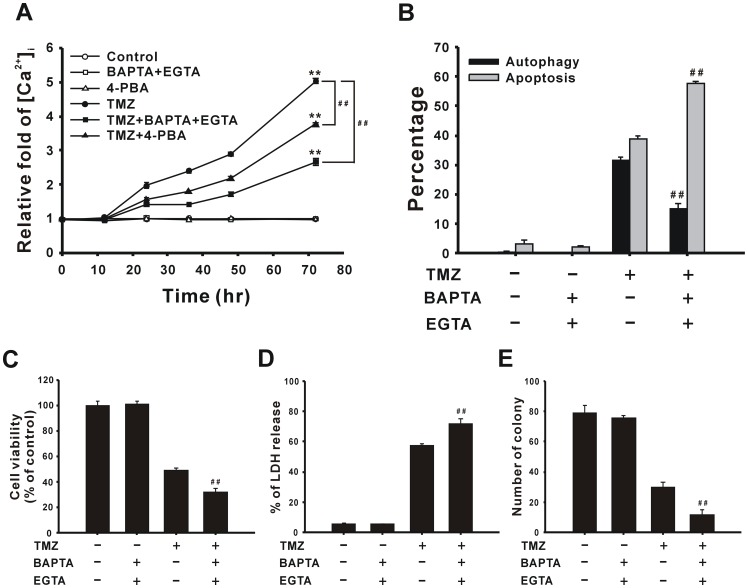
ER stress induces intracellular Ca^2+^ levels after TMZ treatment. (A) U87 MG cells were treated with 400 µM TMZ or combined with 10 mM 4-PBA or 5 µM BAPTA-AM/2 mM EGTA for the indicated time periods. Then the level of intracellular Ca^2+^ was measured using flow cytometry with Fluo-3 AM staining. U87 MG cells were pretreated with the indicated concentrations of 5 µM BAPTA-AM/2 mM EGTA for 1 h, followed by treatment with 400 µM TMZ for another 72 h to determine percentages of cells undergoing autophagy and apoptosis (B), as well as the cell viability (C) and percent of LDH release (D). (E) Colony formation was measured after TMZ and BAPTA-AM/EGTA co-treatment. Results are presented as the mean ± SD. **p<0.01 vs. each respective control. ^##^ *p*<0.01 vs. each respective TMZ group.

ER stress may induce apoptosis or autophagy through different signaling pathways [21]. Therefore, to investigate the downstream signaling of ER stress, we measured the concentration of intracellular calcium ([Ca^2+^]_i_) using flow cytometry with the Fluo-3 AM dye after treatment with 400 µM TMZ for 0–72 h. As shown in Figure 8A, [Ca^2+^]_i_ increased from 24 to 72 h, and was suppressed by 4-PBA, suggesting that the increase of calcium was induced by ER stress. Pretreatment with the calcium chelators, BAPTA-AM and EGTA, also reduced TMZ-induced calcium accumulation (Figure 8A). Moreover, treatment with BAPTA-AM and EGTA decreased and increased TMZ-induced autophagy and apoptosis, respectively (Figure 8B). Ultimately, it was also revealed that cell viability decreased (Figure 8C), and cytotoxicity increased (Figure 8D) with the addition of the calcium chelators according to the MTT and LDH release assays, respectively. After treated cells were grown in soft agar for 3 weeks, the number of colonies was reduced in the group with combined treatment (Figure 8E). These results suggest that ER stress induces calcium accumulation which participates in TMZ-induced autophagy.

**Figure 9:**
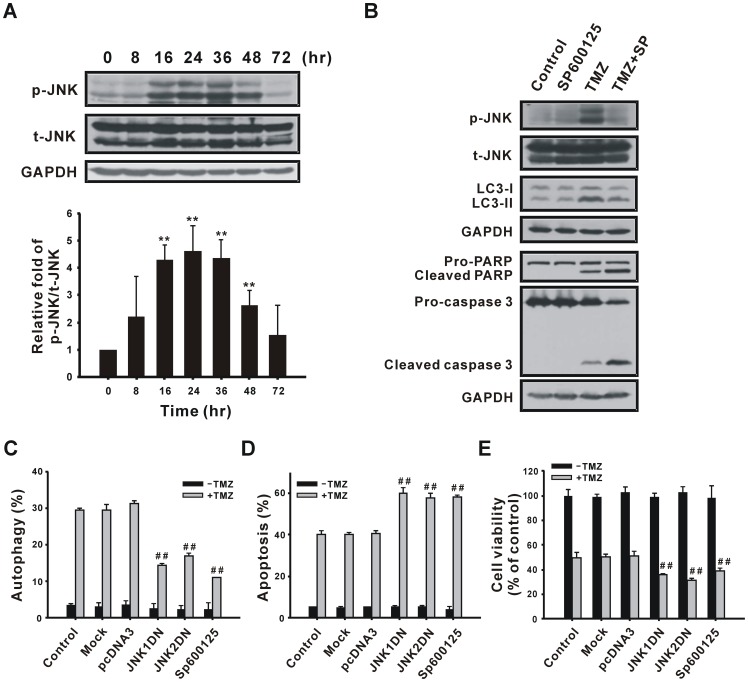
Participation of JNK in TMZ-induced autophagy. (A) U87 MG cells were treated with 400 µM TMZ for the indicated time periods and then analyzed using immunoblotting with anti-p-JNK and anti-t-JNK antibodies. The amount of protein applied in each lane was normalized by t-JNK internal control. (B) U87 MG cells were pretreated with 10 µM SP600125 for 1 h, followed by treatment with TMZ for 36 h (for analysis of JNK and LC3) or 72 h (for analysis of PARP and caspase 3). Cell lysates were analyzed by immunoblotting with anti-p-JNK, anti-t-JNK, anti-LC3, anti-PARP, anti-caspase 3, and anti-GAPDH antibodies. GAPDH and t-JNK were used as internal controls to normalize the amount of protein applied in each lane. U87 MG cells were transfected with 1 µg of the pcDNA3, JNK1DN, or JNK2DN plasmid for 48 h, or pretreated with 10 µM SP600125 for 1 h, followed by treatment with 400 µM TMZ for another 72 h to determine percentages of cells undergoing autophagy (C) and apoptosis (D) as well as cell viability (E). Results are presented as the mean ± SD. t-JNK, total JNK; p-JNK, phosphorylated JNK. **p<0.01 vs. each respective control. *p<0.05, **p<0.01 vs. each respective TMZ group.

To investigate the involvement of JNK in TMZ-induced autophagy, cells were treated with 400 µM TMZ for 0–72 h, and the JNK protein level was detected using immunoblotting. As shown in Figure 9A, TMZ increased the phosphorylation of JNK from 16 to 48 h, which was suppressed by 4-PBA (data not shown). The JNK inhibitor, SP600125, suppressed TMZ-induced JNK phosphorylation and the LC3-II protein, and increased the cleavage of pro-caspase 3 and PARP (Figure 9B), suggesting that TMZ-induced autophagy is mediated by JNK activation. To further confirm the role of JNK in TMZ-induced autophagy, JNK1DN and JNK2DN plasmids were used to down-regulate JNK. Transfection with JNK1DN and JNK2DN or pretreatment with SP600125 to U87 MG cells inhibited the percentage of cells undergoing autophagy (Figure 9C) and increased the percentage of cells undergoing apoptosis (Figure 9D); thus cell viability decreased (Figure 9E). Moerover, the LDH release assay and colony formation assay also indicated that SP600125 increased TMZ-induced cytotoxicity (Figure S2). Taken together, these results suggest that calcium accumulation and JNK activation are required for ER stress-mediated autophagy after treatment with TMZ.

## Discussion

In our previous study, we showed that TMZ treatment increases ROS accumulation, including O_2_^−^, H_2_O_2_, and mtH_2_O_2_ in glioma cells [9]_._ Intracellular ROS are mainly generated from the mitochondrial respiratory chain [10]. With an accumulation of excessive ROS, the activity of the mitochondrial ETC may be influenced [22]. In the present study, we also demonstrated that TMZ induces mitochondrial damage such as mitochondrial depolarization and MPTP opening. These results infer the possibility that TMZ treatment might impact the function of the mitochondrial ETC. Several studies indicated that the mitochondrial respiratory chain may be correlated with agents-induced cell death. For example, the previous study showed that capsaicin-mediated ROS generation inhibited the activities of complexes I and III and disrupted the mitochondrial membrane potential, resulting in caspase-dependent apoptosis in BxPC-3 pancreatic cancer cells. Notably, BxPC-3-derived ρ^0^ cells, which lack normal oxidative phosphorylation, were completely resistant to capsaicin from inducing ROS generation and apoptosis [22]. Oliva et al. revealed that the chemoresistance of TMZ to TMZ-resistant U251 glioma cells or human GBM specimens is due to the remodeled ETC complex activities, including decreases in complexes I and V and increases in complexes II/III and IV. N-Methylmesoporphyrin IX (NMP) treatment increased apoptosis of TMZ-resistant U251 glioma cells through decreasing complex II/III and cytochrome *c* oxidase (complex IV) activities [23]. Compared to our results, the ETC complex IV inhibitor, sodium azide, increased TMZ-induced U87 MG glioma cell death. However, we also demonstrated that chemical inhibition of complex I by rotenone and complex V by oligomycin produced almost the same outcomes in cells. Differences in the above results may have been due to different experimental system.

The onset of the mitochondrial permeability transition (MPT) may initiate the process of autophagy, apoptosis, or necrotic cell death depending on the amount of mitochondria involved [24]. If only a few mitochondria undergo the MPT, then cells can be repaired through the induction of protective autophagy to remove damaged mitochondria [24]. In this study, we demonstrated that TMZ induced mitochondrial damage, including mitochondrial depolarization and MPTP opening, which resulted in induction of protective autophagy. Moreover, inhibition of mitochondrial damage-mediated autophagy by ETC inhibitors or an MPTP inhibitor increased TMZ-induced apoptosis. Likewise, the study of Sy *et al.* showed that the anticancer agent, timosaponin A-III, induced mitochondrial dysfunction, such as oxidative stress, collapse of ΔΨm, and increase of MPTP, leading to protective autophagy to delay mitochondrial-mediated apoptosis in HeLa cells [25]. However, the onset of the MPT induced by metformin or irradiation caused rat C6 glioma and human lung adenocarcinoma cells to undergo apoptosis, respectively [26], [27]. MPT-inducing agents induced apoptosis in TMZ-resistant ADF glioma cells through MPTP opening and ΔΨm dissipation [28]. In addition, tocotrienols caused MPTP-dependent autophagic cell death and apoptosis in rat pancreatic stellate cells [29]. Therefore, those studies suggest that MPTP may individually or simultaneously induce apoptosis and/or autophagy to regulate the cell fate.

Mitochondria are also the target of autophagy, and the selective degradation of mitochondria through autophagy is called mitophagy [4]. Mitophagy in remodeling rat hepatocytes leaded to a decrease in the mitochondrial number and mass [4]. CsA inhibition of MPTP-dependent autophagy resulted in higher levels of mitochondrial proteins and contents in starved cardiac cells [30]. Therefore, autophagy can eliminate dysfunctional mitochondria. In our study, the combination of 3-MA and TMZ increased mitochondrial mass, indicating that mitochondria-mediated autophagy triggered the loss of mitochondrial mass in U87 MG glioma cells. This may be the reason that the opening of MPTP increased in an early time period but declined in a later stage after treatment with TMZ, since the damaged mitochondria were removed through autophagy. However, determining whether TMZ-induced autophagy is truly mitophagy requires further investigation. A possible mechanism of how mitochondrial autophagy can protect cells was proposed of smaller amounts of pro-apoptotic molecules possibly being released from mitochondria [2]. Yang *et al.* also reported that MPT-mediated mitochondrial autophagy was induced after heat shock to inhibit both cytochrome *c* release and caspase 3 activation, thus protecting cells from apoptosis [31]. Determining whether pro-apoptotic molecules such as cytochrome *c* are released from mitochondria to enhance apoptosis after TMZ treatment requires further study. However, the previous study indicated that the generation of superoxide anion induced by 7 µM selenite treatment triggered disruption of mitochondria, resulting in mitophagy and subsequent nonapoptotic cell death in glioma cells [32]. Therefore, those results suggest that mitochondria-mediated autophagy can lead to either cell survival or death.

ER stress may result in cell dysfunction or cell death under various stressful conditions [16], [33]. In a previous study of our group, cadmium induced ER stress-mediated apoptosis in mesangial cells [34]. Although ER stress can trigger apoptosis, it also has a pro-survival role under different stress conditions. ER stress initiates the UPR and transduces signaling pathways to restore normal functions [16]. A previous study showed that cells initiated a self-protective response during mild ER stress [18]. For instance, ER stress inducers (thapsigargin and tunicamycin) protected SH-SY5Y cells from 6-hydroxydopamine (6-OHDA)-induced cytotoxicity [35]. In this study, we revealed that TMZ induced ER stress as indicated by the increased protein levels of GADD153 and GRP78 and decreased level of caspase12 (Figure 7A and B). Suppression of ER stress by the chemical chaperone, 4-PBA, reduced TMZ-induced autophagy and cell viability (Figure 7D and E), which indicated that the pro-survival role of ER stress acts through inducing protective autophagy in glioma cells. Although activation of caspase 12 demonstrates that TMZ induces ER stress-mediated apoptosis, the combination of 4-PBA increased TMZ-induced apoptosis and cleavage of PARP and caspase 3 (Figure 7D and E), suggesting that the effect of ER stress-mediated autophagy on cell fate is more important than ER stress-mediated apoptosis. Our data are consistent with the findings of other studies that ER stress-mediated autophagy promotes survival of atorvastatin-treated hepatocellular and colorectal carcinoma cells [36] or 2-deoxy-D-glucose-treated pancreatic cancer and melanoma cells [37]. Pyrko’s study indicated that knockdown of GRP78 sensitized glioma cells to TMZ [38]. Besides, the addition of epigallocatechin gallate (EGCG) enhanced the therapeutic effect of TMZ by inhibiting GRP78 function [39]. Therefore, those studies and ours suggest that inhibition of ER stress may be a strategy to enhance the cytotoxicity of TMZ.

ER stress triggers autophagy or apoptosis through either PERK/eIF2α, IRE1/TRAF2/JNK, or Ca^2+^ signaling pathways [21]. Herein, we demonstrated that TMZ-induced ER stress leads to both cytosolic Ca^2+^ accumulation and JNK activation, thereby promoting the process of autophagy to protect U87 MG glioma cells from death. ER stress induced by tunicamycin and capsaicin also triggered IRE1/JNK- and eIF2α-dependent autophagy to promote the survival of SK-N-SH neuroblastoma cells and WI-38 cells, respectively [19], [40]. In other cases, ER stress mediated radiation- or glucosamine-induced autophagic cell death through the phosphorylation of eIF2α [41], [42]. In our previous study, ER stress induced by cadmium caused an elevation of the cytosolic Ca^2+^ concentration leading predominantly to autophagic cell death and a minor level of apoptotic cell death [34]. Therefore, ER stress may cause autophagy to either protect or kill cells in different environments.

**Figure 10:**
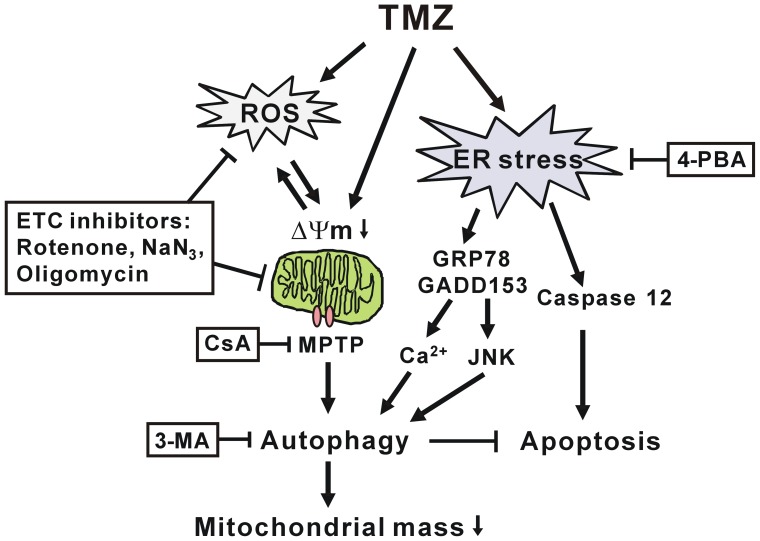
A schematic model of TMZ-induced autophagy and apoptosis. As described in detail in the text, TMZ induces ROS accumulation, mitochondrial depolarization, and MPTP opening to cause mitochondrial damage, which result in the process of protective autophagy. ETC inhibitors can reverse the above phenomena, thus enhancing TMZ-induced apoptosis. In addition, TMZ triggers ER stress with changes of GRP78, GADD153, and caspase 12 proteins. ER stress also induces autophagy through the action of JNK and intracellular calcium accumulation in glioma cells.

In conclusion, we demonstrated that TMZ induces mitochondrial damage, such as ROS generation, mitochondrial depolarization, and MPTP opening, resulting in protective autophagy to decrease mitochondrial mass. TMZ treatment also triggers ER stress-mediated autophagy and apoptosis. Inhibitors of the mitochondrial ETC, MPTP, or ER stress are able to reduce autophagy and consequently increase TMZ-induced apoptosis (Figure 10). Our results may provide advantageous insights for development of suitable therapies for brain tumors by targeting mitochondria and ER.

## Materials and Methods

### Reagents and Antibodies

Minimum essential medium (MEM) and supplements were purchased from Hyclone (Logan, UT). The JNK1 dominant-negative mutant (DN) and JNK2 DN were provided by kindly Dr. CH Lin (Taipei Medical University, Taipei, Taiwan) [43]. CsA, SP600125, 4-PBA, and 3-(4,5-dimethyl-2-thiazolyl)-2,5-dimethyl-2H-tetrazolium bromide (MTT) were purchased from Merck (Darmstadt, Germany). TMZ, acridine orange, 3-MA, rhodamine 123, CoCl_2_, rotenone, sodium azide, oligomycin, BAPTA-AM, EGTA, and crystal violet were purchased from Sigma (St. Louis, MO). Fluo-3 AM, DCFH-DA, HEt, and DHR123 were purchased from Molecular Probes (Eugene, OR). A protease inhibitor cocktail was purchased from Roche (Boehringer, Mannheim, Germany). Polyvinylidene difluoride (PVDF) membranes were purchased from Millipore (Bedford, MA). The Protein Assay Dye Reagent was obtained from Bio-Rad Laboratories (Hercules, CA). Phenol red-free RPMI 1640 medium, penicillin/streptomycin, sodium pyruvate, calcein AM, Fluo3-AM, Hank’s balanced salt solution (HBSS, containing calcium), and NAO were purchased from Invitrogen (Carlsbad, CA). Annexin V was obtained from Biovision (Mountain View, CA). Propidium iodide (PI) was purchased from Calbiochem (San Diego, CA). Anti-LC3 and GADD153 antibodies were purchased from NOVUS (Littleton, CO); anti-glyceraldehyde 3-phosphate dehydrogenase (GAPDH), p-JNK, JNK, poly (ADP-ribose) polymerase (PARP), caspase 12, and caspase 3 antibodies were purchased from Cell Signaling Technology (Beverly, MA); the anti-caspase 12 antibody was purchased from Abcam (Cambridge, MA); and the anti-GRP78 antibody was purchased from BD Biosciences (Sparks, MD). The secondary antibodies, including horseradish peroxidase (HRP)-conjugated goat anti-mouse and anti-rabbit immunoglobulin G (IgG), were purchased from Jackson ImmunoResearch (West Pine, PA).

### Cell Culture

The human glioblastoma U87 MG cell line classified as grade IV was purchased from the Bioresource Collection and Research Center (BCRC, HsinChu, Taiwan). U87 MG cells were maintained in MEM and supplemented with 10% fetal bovine serum (FBS), 100 units/ml penicillin, 100 µg/ml streptomycin, 1 mM sodium pyruvate and 1 mM nonessential amino acids at 37°C in a 5% CO_2_ incubator.

### Cell Viability Assay

Cells were seeded on a 96-well plate at 8×10^3^ cells/well for 24 h, followed by individual treatments for the indicated time periods. Before the end of treatment, 0.5 mg/ml MTT was added to each well for 4 h. The supernatants were carefully aspirated, and the formazan crystals were dissolved in DMSO. The absorbance was measured at 550 nm with a Thermo Varioskan Flash reader (Thermo Electron Corporation, France).

### LDH Release Assay

U87 MG cells were seeded on a 96-well plate for 24 h, followed by individual treatments for the indicated time periods. At the end of treatment, LDH release was measured using a CytoTox 96® Non-Radioactive Cytotoxicity Assay Kit (Promega) according to the manufacturer’s protocol. LDH release induced by lysis buffer supplied by the manufacturer was set to 100%.

### Colony Formation Assay

U87 MG cells (1×10^3^) were mixed in culture medium with 0.25% low-melting point agarose and poured on 0.5% base agarose/medium in 6-well plates. Medium containing TMZ and/or the indicated drugs was added in triplicate to the appropriate wells. Cells were incubated for 3 weeks at 37°C in a 5% CO_2_ incubator, and the medium was changed every 3 to 4 days. At the end of the experiment, colonies were stained with crystal violet and counted. Three independent experiments were done.

### Detection of Autophagy

Autophagy is characterized by the formation and promotion of acidic vesicular organelles (AVOs) [44]. Flow cytometry with acridine orange staining was employed to detect and quantify the AVOs. In acridine orange-stained cells, the cytoplasm and nucleus fluoresce bright green and dim red, whereas acidic compartments fluoresce bright red, as described previously [44]. Therefore, we could measure a change in the intensity of the red fluorescence to represent the percentage of cellular acidic compartments. After treatment with TMZ alone or TMZ combined with other drugs, 1×10^5^ cells were collected in phenol red-free RPMI 1640 medium. The green (FL-1) and red (FL-3) fluorescences of acridine orange were measured with a flow cytometer using CellQuest software (Becton Dickinson, San Jose, CA). The sum of the upper-left and upper-right quadrants of the cytogram was used to represent the percentage of cells undergoing autophagy.

### Detection of Apoptosis

Apoptosis was analyzed using flow cytometry with annexin V/propidium iodide (PI) double-staining to detect membrane events [45]. In brief, after treatment with various drugs in different experiments, whole cells were collected in HEPES buffer containing 10 mM HEPES (pH 7.4), 140 mM NaCl, and 2.5 mM CaCl_2_. Subsequently, cells were stained with annexin V (2.5 µg/ml) and PI (2 ng/ml) for 20 min, followed by analysis on a flow cytometer using CellQuest software. The cytogram of the four quadrants in the figure was used to distinguish normal (annexin V^−/^PI^−^), early apoptotic (annexin V^+^/PI^−^), late apoptotic (annexin V^+^/PI^+^), and necrotic cells (annexin V^−/^PI^+^). The sum of early and late apoptosis was presented as total apoptosis [45].

### Measurement of the Mitochondrial Membrane Potential

The mitochondrial membrane potential was detected by flow cytometry with rhodamine 123 staining. Briefly, U87 MG cells were treated with 400 µM TMZ for various time periods, and then cells were collected by trypsinization. Cells were stained with 1 µM rhodamine 123 in the dark at 37°C for 30 min, and the density of green fluorescence was analyzed with flow cytometry. The percentage of cells with decreased fluorescence was counted as the degree of collapse of mitochondrial membrane potential.

### Detection of the Mitochondrial Permeability Transition Pore (MPTP) Opening

The opening of MPTP was examined using calcein AM staining combined with CoCl_2_ to detect the mitochondrial calcein fluorescence [46]. Calcein AM freely passes though cellular membranes, and the esterases cleave the acetomethoxy group to yield the fluorescent calcein which is trapped inside cells. Co-loading of cells with CoCl_2_ quenches the fluorescence in the cell, except in mitochondria, since CoCl_2_ cannot cross mitochondrial membranes. Therefore, during the opening of MPTP, mitochondrial calcein is also quenched by CoCl_2_, resulting in reduced fluorescence. At the end of treatment, cells were trypsinized and incubated at 37°C in 1 ml HBSS containing calcium (HBSS/Ca) with 1 µM calcein AM and 5 mM CoCl_2_ for 20 min. After incubation, the fluorescence of mitochondrial calcein was analyzed by flow cytometry.

### Determination of Mitochondrial Mass

The mitochondrial mass was measured by NAO which can bind to cardiolipin at the inner membrane of mitochondria in a membrane potential-independent manner regardless energization [47], [48]. At the end of treatment, cells were trypsinized and resuspended in 1 ml PBS containing 0.5 µM NAO for 15 min at 37°C in the dark. Then, cells were immediately analyzed by flow cytometry. The mean values of NAO fluorescence were normalized to that of control cells.

### Measurement of ROS

Intracellular levels of H_2_O_2_ and O_2_^−^ were respectively detected using the probes DCFH-DA and HEt. DCFH-DA and HEt are oxidized by H_2_O_2_ and O_2_^−^, and then emit green and red fluorescence, respectively. Furthermore, the probe DHR123 can enter mitochondria, and the green fluorescence represents the mitochondrial H_2_O_2_ level [49]. Glioma cells were collected at appropriate time points after different treatments. After trypsinization, cells were resuspended and stained with 10 µM DCFH-DA, 5 µM HEt, or 5 µM DHR123 for 30 min at 37°C in the dark. Fluorescence was measured on a flow cytometer using CellQuest software. The percentage of increase in fluorescence peak was used to represent the level of ROS production.

### Immunoblotting

Cells were lysed with lysis buffer (25 mM HEPES, 1.5% Triton X-100, 0.1% sodium dodecylsulfate (SDS), 0.5 M NaCl, 5 mM EDTA, and 0.1 mM sodium deoxycholate) containing a protease inhibitor cocktail. Total cellular extracts were analyzed using the Bio-Rad protein assay dye reagent, and an equal amount of proteins from each group was separated using SDS-polyacrylamide gel electrophoresis (PAGE), followed by transfer to PVDF membranes. Membranes were incubated with a 5% skim milk solution (blocking solution) for 1 h, and then incubated with the indicated antibodies at 4°C for 16 h. Membranes were probed with appropriate HRP-conjugated secondary antibodies for 1 h at room temperature. Immunoreactive proteins were detected using an enhanced chemiluminescence reagent (SuperSignal West Pico Chemiluminescent Substrate, Pierce Biotechnology, Rockford, IL) and then exposed to X-ray film (FUJIFILM Corporation, Tokyo, Japan). The density of bands was determined with Gel-Pro Analyzer densitometry software.

### Measurement of Intracellular Calcium

At the end of treatment, U87 MG cells were harvested and incubated with 500 nM Fluo-3 AM dye for 30 min at 37°C. Then, cells were collected and analyzed on a flow cytometer using FL-1 as a detector. The relative intracellular calcium concentrations were calculated from the geographic mean values of the FL-1 peak and were presented as the ratio compared to each respective control.

### Transfection

U87 MG cells (5×10^4^ cells/well) were seeded onto 6-well plates and transfected with 1 µg pcDNA3, JNK1DN, or JNK2DN using the Lipofectamine2000 reagent (Invitrogen) according to the manufacturer’s protocol. After transfection, cells were treated with 400 µM TMZ for 72 h, and cell viability and the percentages of autophagy and apoptosis were determined.

### Statistical Analysis

Data are presented as the mean ± standard deviation (SD) from three independent experiments. Statistical significance was examined using Student’s *t*-test for comparing paired sample sets. A *p* value of <0.05 was considered statistically significant.

## Supporting Information

Figure S1 **Effects of autophagy inducer, rapamycin, on TMZ-treated U87 MG glioma cells.** U87 MG cells were pre-treated with or without 0.5 µM rapamycin for 1 h followed by incubation with 400 µM TMZ for 72 h to determine the percentages of cells undergoing autophagy (A) and apoptosis (B), as well as cell viability (C). Results are presented as the mean ± SD. **p<0.01 vs. each respective TMZ group.
(TIF)

Figure S2 **Effect of SP600125 on TMZ-induced cytotoxicity.** U87 MG cells were treated with 10 µM SP600125 and 400 µM TMZ for 72 h or 21 days, and were supplied to LDH release assay (A) and soft agar colony formation assay (B), respectively. *p<0.05, **p<0.01 vs. each respective TMZ group.
(TIF)
