# Supplementary figures and images for: Inhibition of Mitochondria- and Endoplasmic Reticulum Stress-Mediated Autophagy Augments Temozolomide-Induced Apoptosis in Glioma Cells
Source: PLoS One. 2012 Jun 22;7(6):e38706. doi: 10.1371/journal.pone.0038706 (PMC3382156; doi:10.1371/journal.pone.0038706)

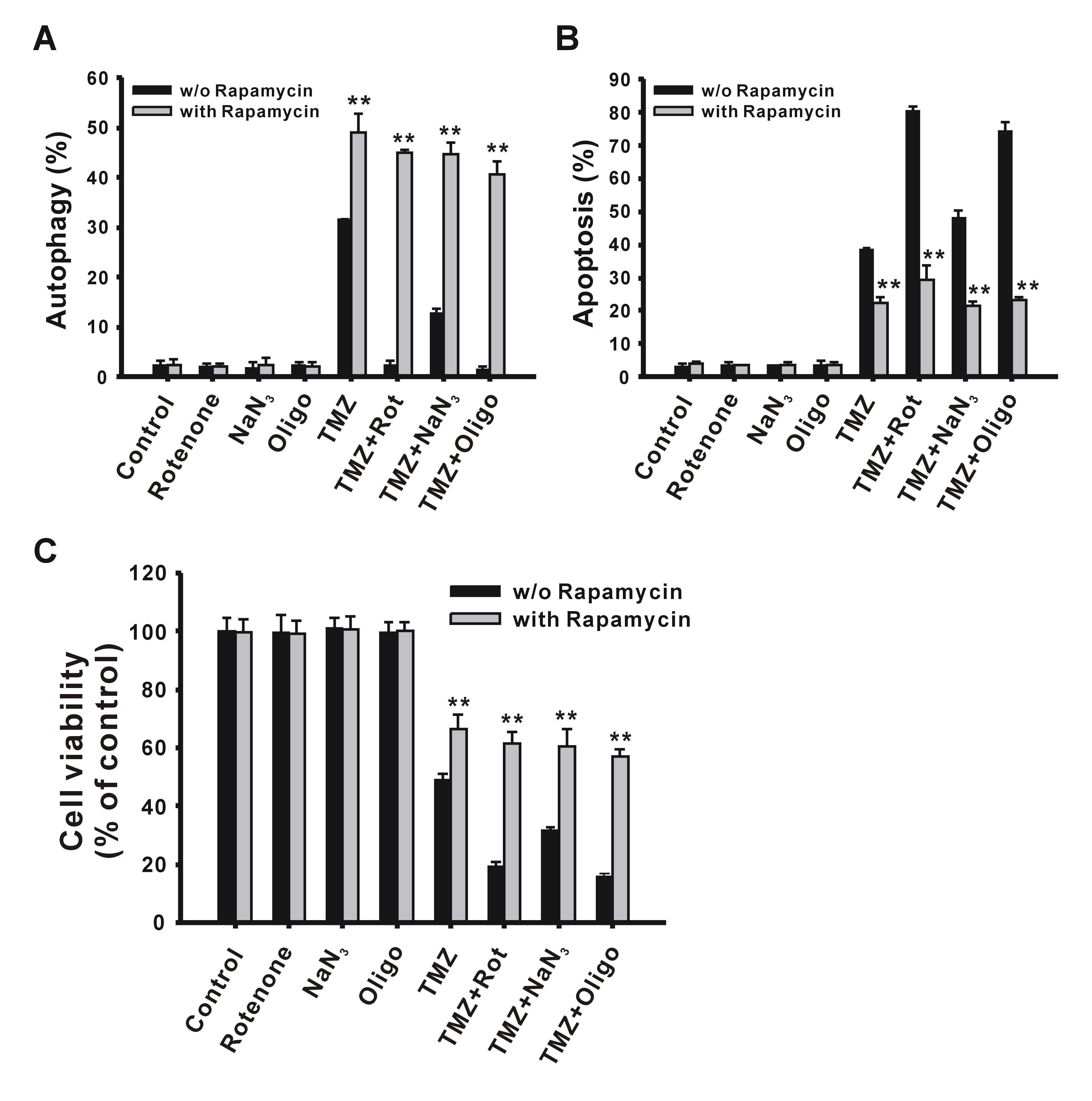

Supplement: Figure S1 — Effects of autophagy inducer, rapamycin, on TMZ-treated U87 MG glioma cells. U87 MG cells were pre-treated with or without 0.5 µM rapamycin for 1 h followed by incubation with 400 µM TMZ for 72 h to determine the percentages of cells undergoing autophagy (A) and apoptosis (B), as well as cell viability (C). Results are presented as the mean ± SD. **p<0.01 vs. each respective TMZ group. (TIF) [file pone.0038706.s001.tif]

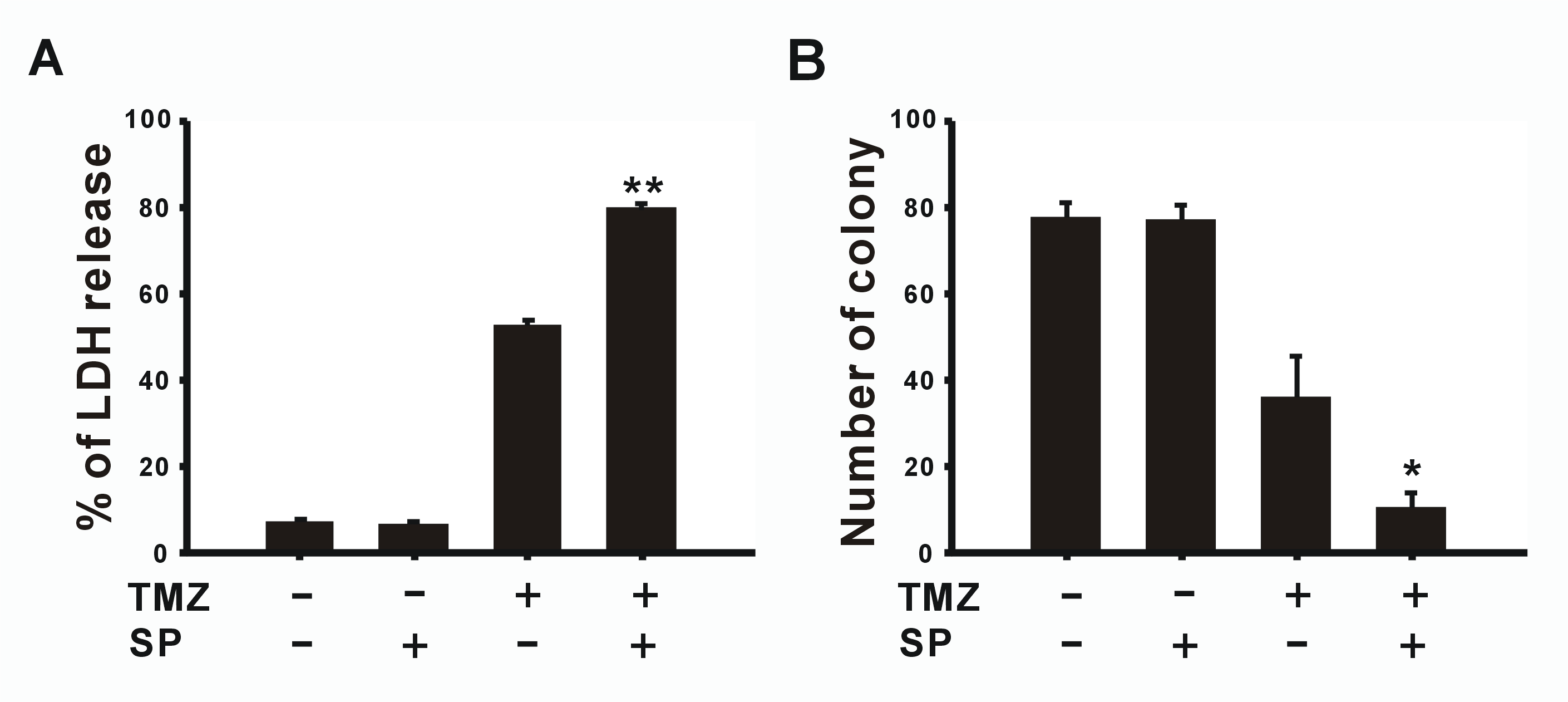

Supplement: Figure S2 — Effect of SP600125 on TMZ-induced cytotoxicity. U87 MG cells were treated with 10 µM SP600125 and 400 µM TMZ for 72 h or 21 days, and were supplied to LDH release assay (A) and soft agar colony formation assay (B), respectively. *p<0.05, **p<0.01 vs. each respective TMZ group. (TIF) [file pone.0038706.s002.tif]
